# Using proteomic analysis to investigate uniconazole-induced phytohormone variation and starch accumulation in duckweed (*Landoltia punctata*)

**DOI:** 10.1186/s12896-015-0198-9

**Published:** 2015-09-15

**Authors:** Mengjun Huang, Yang Fang, Yang Liu, Yanling Jin, Jiaolong Sun, Xiang Tao, Xinrong Ma, Kaize He, Hai Zhao

**Affiliations:** Chengdu Institute of Biology, Chinese Academy of Sciences, Chengdu, 610041 China; University of Chinese Academy of Sciences, Beijing, 100049 China; Key Laboratory of Environmental and Applied Microbiology, Chinese Academy of Sciences, Chengdu, 610041 China; Environmental Microbiology Key Laboratory of Sichuan Province, Chengdu, 610041 China

## Abstract

**Background:**

Duckweed (*Landoltia punctata*) has the potential to remediate wastewater and accumulate enormous amounts of starch for bioethanol production. Using systematical screening, we determined that the highest biomass and starch percentage of duckweed was obtained after uniconazole application. Uniconazole contributes to starch accumulation of duckweed, but the molecular mechanism is still unclear.

**Results:**

To elucidate the mechanisms of high starch accumulation, in the study, the responses of *L. punctata* to uniconazole were investigated using a quantitative proteomic approach combined with physiological and biochemical analysis. A total of 3327 proteins were identified. Among these identified proteins, a large number of enzymes involved in endogenous hormone synthetic and starch metabolic pathways were affected. Notably, most of the enzymes involved in abscisic acid (ABA) biosynthesis showed up-regulated expression, which was consistent with the content variation. The increased endogenous ABA may up-regulate expression of ADP-glucose pyrophosphorylase to promote starch biosynthesis. Importantly, the expression levels of several key enzymes in the starch biosynthetic pathway were up-regulated, which supported the enzymatic assay results and may explain why there is increased starch accumulation.

**Conclusions:**

These generated data linked uniconazole with changes in expression of enzymes involved in hormone biosynthesis and starch metabolic pathways and elucidated the effect of hormones on starch accumulation. Thus, this study not only provided insights into the molecular mechanisms of uniconazole-induced hormone variation and starch accumulation but also highlighted the potential for duckweed to be feedstock for biofuel as well as for sewage treatment.

**Electronic supplementary material:**

The online version of this article (doi:10.1186/s12896-015-0198-9) contains supplementary material, which is available to authorized users.

## Background

Renewable biofuels, such as bioethanol, derived from biomass is considered to be the most promising alternative to petroleum liquid fuels. The development of bioethanol can reduce greenhouse gas emissions and meet the strong global demand for energy [1]. Current commercial production of ethanol fuel has focused on using starch and sugar from maize, sugarcane [2], cassava [3], and sweet potato [4]. However, there is some controversy surrounding these feedstocks that often compete with food crops for arable land [5, 6]. For the most abundant lignocellulosic sources, there is still a lack of an economical, efficient, and environmentally beneficial pretreatment process for ethanol biofuel production [7]. Therefore, exploration and research of novel alternative resources for bioethanol production has attracted tremendous interest.

Duckweed, classified as a separate family *Lemnaceae* earlier, arose from the arum or aroid family [8] and therefore, often are classified as the subfamily *Lemnoideae* within the *Araceae*. As a global adaptable aquatic macrophyte [9], the plant is a potential alternative feedstock for bioethanol production [10–14] because of its high starch accumulation and very low lignin percentage [15]. Duckweed has been used for wastewater treatment such as domestic sewage and industrial wastewater; the biomass generated was also sufficient for biofuels production while removing nutrients [16, 15, 17, 18]. The relative growth rate of duckweed is 12 g/m^2^/day dry weight (DW) in warm regions [19]; the average annual rate is 7.26 g DW/m^2^/day, enough to yield 26.50 tons per hectare, indicating that duckweed has a higher yield than most terrestrial plants [20]. By manipulating the growth conditions, such as pH, phosphate concentration, nutrient starvation, and light conditions, duckweed can accumulate starch up to 52.9 % of its DW [10, 12, 21, 22]. Because of these key attributes for a potential biofuel feedstock, duckweed has received much attention from academia, industry, and the government. At present, there is some pilot-scale research on duckweed and its applications [23, 24].

Plant growth regulators (PGRs) have been widely used to manipulate plant growth and the yield of crops [25, 26]. To enhance the biomass and starch accumulation of duckweed for bioethanol fermentation under large-scale cultivation, we also used PGRs to regulate duckweed growth. Approximately twenty PGRs were screened systematically at different concentration gradients. One of the main findings was that uniconazole applied as spraying the surface of a population at 800 mg/L yielded the highest biomass and starch content of duckweed. Uniconazole, an active member of the triazole family, has been used in agriculture to enhance stress tolerance [27, 28] and grain quality of rice [29] and wheat [30] by inhibition of gibberellin (GA) biosynthesis [25]. Similarly, it has significantly increased the starch content of potato tubers by approximately 8 % [31] and starch grain accumulation in lotus rhizomes [32]. Plants respond to uniconazole through various biochemical and physiological processes, but the molecular mechanism is still unclear. Whether or not the uniconazole-induced starch accumulation mainly depends on endogenous GA, is still unknown. There has thus far been little research linking uniconazole with expression changes in endogenous hormone biosynthesis enzymes and on the roles of certain hormone variations that cause high starch accumulation.

Bioinformatics, such as transcriptomics and proteomics, are useful as they could provide global information about gene expression. These methods could be powerful to analyze the metabolic pathways of non-model plants, since it is difficult to elucidate the metabolic mechanism without genetic transformation system. The protein, as the functional executor, is most closely related to physiological changes. Hence, it is more important to investigate downstream protein expression by proteomic technology. Our previous proteomic study of *L. punctata* has reported its high starch accumulation and low lignin percentage under nutrient starvation [33]. However, how duckweed accumulates high levels of starch with uniconazole application is still unclear. In the present study, the proteomic analysis of the uniconazole-treated duckweed was performed with isobaric tags for the relative and absolute quantification (iTRAQ) technique [34]. These results provide important information about the molecular mechanisms of hormone changes and starch accumulation with uniconazole application, which can further develop duckweed as a bioenergy crop.

## Results and discussion

### Proteomic research of *L. punctata* after application of uniconazole

The establishment of a *L. punctata* database based on transcriptome data contributed to protein identification. In our study, a total of 369,230 spectra were obtained from iTRAQ liquid chromatography-tandem mass spectrometry (LC-MS/MS) proteomic analysis. Because the whole genome of *L. punctata* has not yet been completely identified, a protein sequence database was established using the mRNA transcripts derived from our RNA-Seq data of *L. punctata* under uniconazole treatment. The translated mRNA sequence database was generated by translation of all open reading frames (ORFs) for these transcripts by 6-frame translation [35]. After searching against the database, 40,996 unique spectra that met strict identification criteria were matched to 13,457 unique peptides and 3327 proteins.

**Fig. 1:**
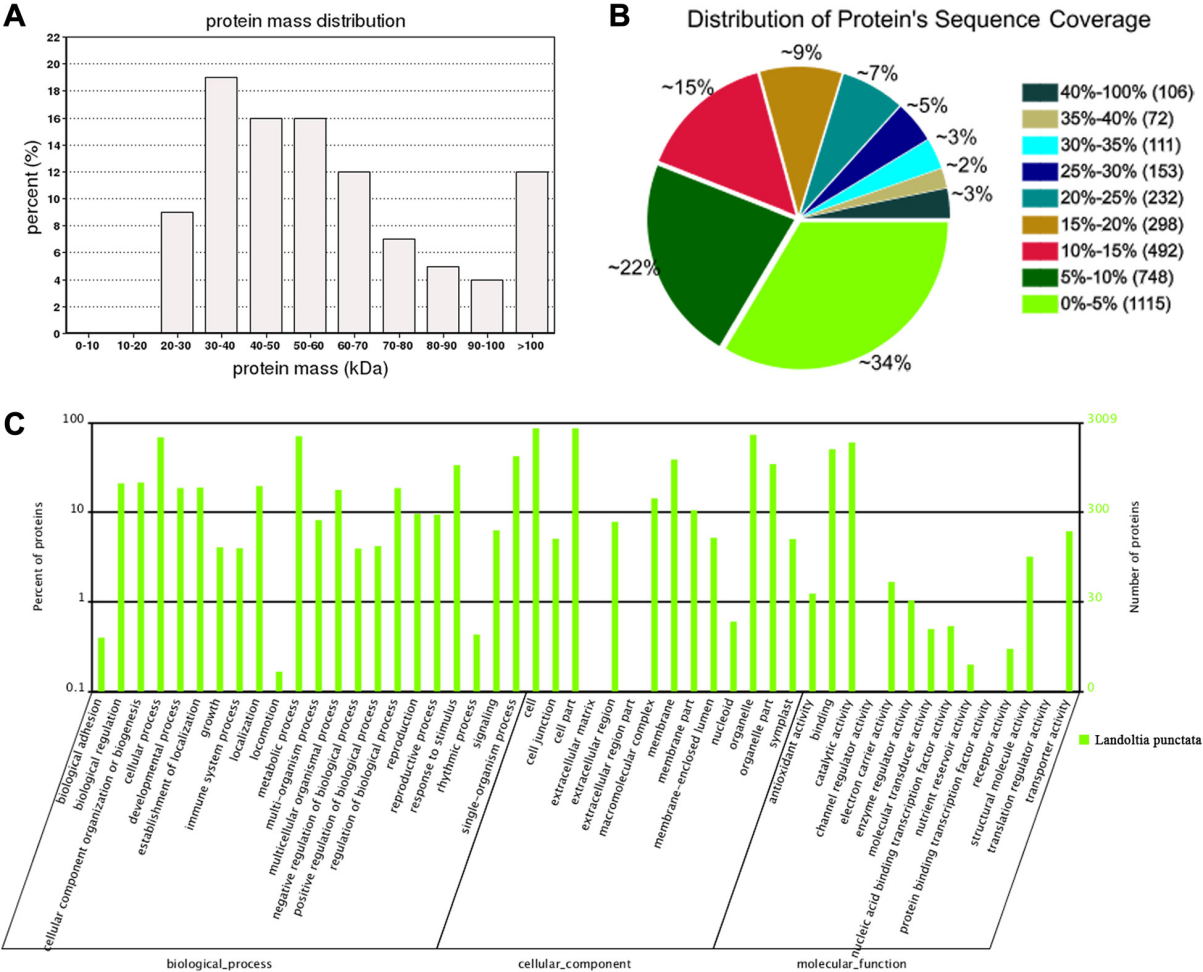
Distribution, coverage, and functional category of proteins identified in the study. **a** Distribution of identified proteins among different molecular weights; (**b**) Coverage of proteins by the identified peptides; (**c**) Functional category of identified proteins

In terms of protein molecular weight distribution, there was very good coverage (all proteins) for a wide range for proteins larger than 20 kDa (Fig. 1a). In addition, most of the proteins were identified with good sequence coverage; approximately 66 % of the proteins were with more than 5 % of the sequence coverage, and approximately 44 % were with 10 % of the sequence coverage (Fig. 1b). All identified unique proteins were classified into three ontologies: biological process, cellular component, or molecular function (Fig. 1c). The main subcategories within the cellular component proteins were cell (23.52 %) and cell part (23.52 %). In the molecular function category, the most frequently detected gene ontology (GO) terms included catalytic activity and binding, representing 47.86 and 40.22 %, respectively. The biological process category was mainly represented by metabolic process and cellular process proteins, representing 17.62 and 17.09 %, respectively. Using a cutoff of a fold change >1.2 or <0.8 with a *p*-value of less than 0.05, 969 proteins were quantified as having significant changes in expression. There were 578 proteins that had increased abundance (Additional file 1) and 391 that had decreased (Additional file 2) after uniconazole application. The proteins that had differential expression among different time points are listed in the Table 1. Among these proteins, 420 proteins were found to be responsive to uniconazole between 240 and 0 h (Table 1).

**Table 1:** Proteomic changes in duckweed among different time points

Comparisons	Up-regulated proteins (No.)	Down-regulated proteins (No.)	Total (No.)
2 h vs 0 h	17	9	26
5 h vs 0 h	8	7	15
72 h vs 0 h	142	75	217
240 h vs 0 h	273	147	420
5 h vs 2 h	8	26	34
72 h vs 2 h	117	96	213
240 h vs 2 h	221	162	383
72 h vs 5 h	130	66	196
240 h vs 5 h	236	146	382
240 h vs 72 h	104	50	154

Numbers of proteins that had differential expression were listed in the table

Metabolic pathway enrichment analysis was performed for the differentially displayed proteins to determine the affected cellular metabolism. These proteins were matched to the proteins annotated with the KEGG pathway database, and the frequencies of the responsive proteins in each KEGG pathway were compared to determine statistical authenticity of the involvement of that pathway induced by uniconazole. At 2, 5, 72, and 240 h after spraying uniconazole, 21, 12, 65, and 94 KEGG pathways were affected, respectively, with *p*-values less than 0.05. Carotenoid biosynthesis (KO00906) and starch and sucrose metabolism (KO00500) were also enriched (Additional file 3), suggesting that these proteins in these pathways were highly expressed and may be active during the treatment condition. These pathways are very important for hormone variation and starch accumulation, which are our main focuses. Hence, the expression profiles of these enzymes involved in relevant pathways need more investigation.

The establishment of a protein sequence database based on transcriptomic data is suitable for proteomic analysis of species that have not yet been whole-genome sequenced. There were also successful cases in *Bugula neritina* [36], *Macleaya cordata*, and *Macleaya microcarpa* [37], and *L. punctata* under nutrient starvation from our previous proteomic study [33]. There was a high concordance in variation trends of enzymes when comparing protein with transcript in response to uniconazole. On the one hand, most of the identified enzymes involved in hormone biosynthesis, such as zeaxanthin epoxidase (EC: 1.14.13.90; ZEP) and 9-cis-epoxycarotenoid dioxygenase (EC: 1.13.11.51; NCED) (Additional file 1), were consistent with the transcripts in terms of trends. The up-regulated expression of these enzymes contributed to ABA biosynthesis. On the other hand, many key enzymes in the starch metabolic pathway, such as ADP-glucose pyrophosphorylase (EC: 2.7.7.27; AGP) and granule bound starch synthase (EC: 2.4.1.11; GBSS) shared the same up-regulated expression direction at both the protein and transcript levels. Their expression contributes to rapid accumulation of high levels of starch. However, some enzymes displayed different trends between proteome and transcriptome. For example, the transcript expression of soluble starch synthetase (EC: 2.4.1.21; SSS) decreased, but the protein expression displayed no significant change. Several reasons may account for this variation. First, there are various expression orders and abundance changes at different phases. These processes may also include posttranscriptional, translational and/or posttranslational regulation [38]. Second, the shortage of genome information also affects identification of some unique proteins. Moreover, existing techniques have limitations in identification and quantification at the two levels. A similar conclusion was also obtained by our earlier study with transcriptomics [39] and proteomics data [33] collected from *L. punctata* under nutrient starvation. Importantly, our time-series proteomic results can provide a more direct expression pattern than other methods, which also supported the physiological changes after application of uniconazole.

### Phytohormone change of *L. punctata* under uniconazole treatment

**Fig. 2:**
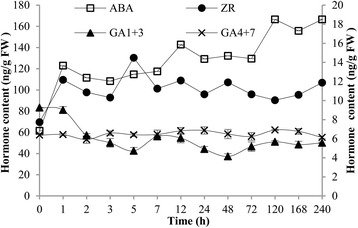
Effect of uniconazole on endogenous hormone levels of duckweed. The ABA content corresponds to the main Y-axis, the contents of ZR, GA1 + 3 and GA1 + 4 correspond to the minor Y-axis

The contents of abscisic acid (ABA) and zeatin-riboside (ZR) increased, but the gibberellin (GA) content decreased with uniconazole application. ABA had the highest content among these measured phytohormones of all samples. There was a significant increase in ABA content from 61.47 to 166.53 ng/g FW at 240 h (Fig. 2). Similarly, ZR, a type of cytokinin, also slightly increased from 7.73 to11.87 ng/g FW by 240 h. On the contrary, GA_1+3_ content decreased from 9.25 to 5.57 ng/g FW at 240 h while GA_4+7_ content maintained a stable low level.

**Fig. 3:**
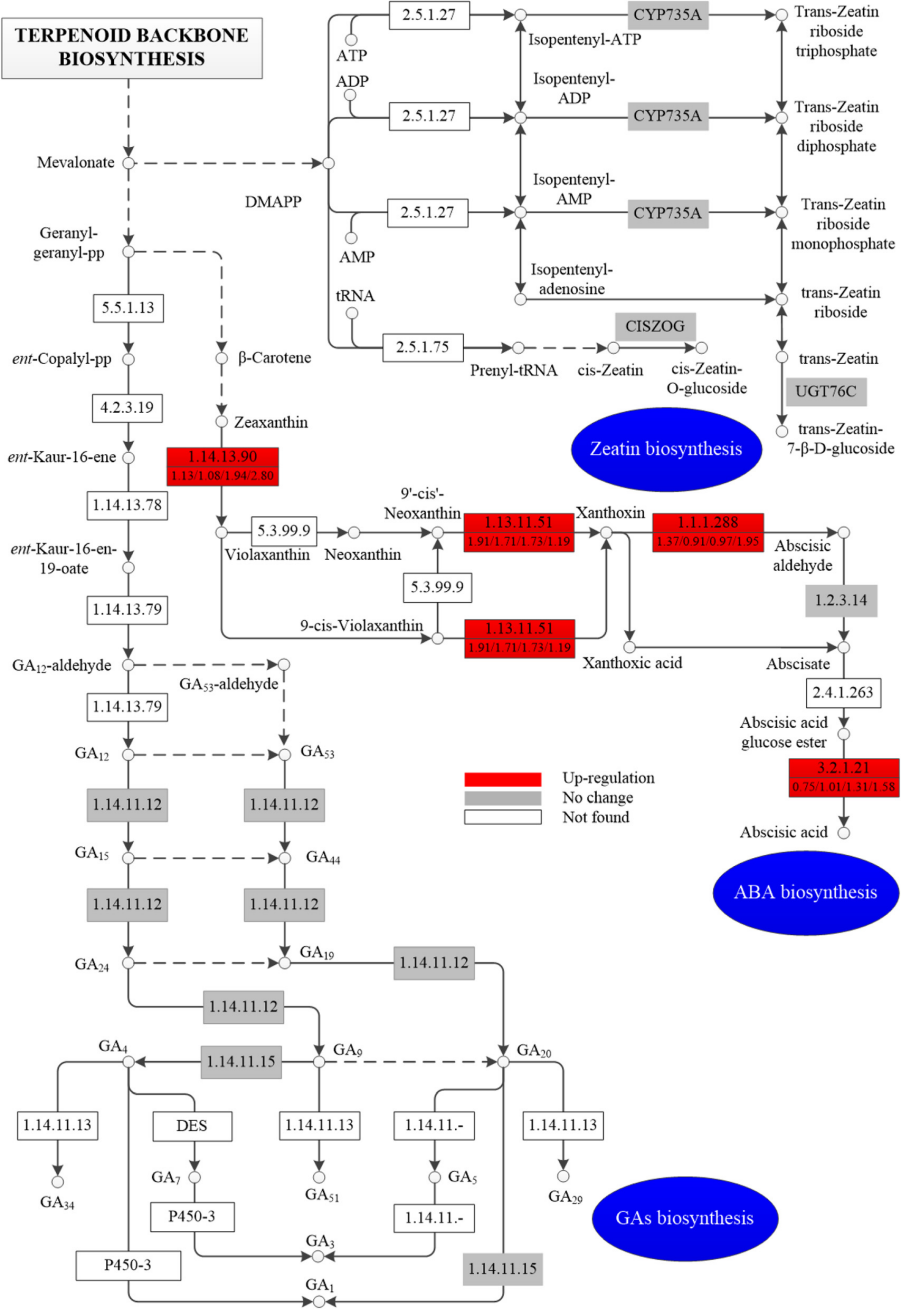
Expression patterns of some enzymes involved in hormones biosynthesis. Red boxes indicate the up-regulated enzymes in response to uniconazole, gray means no significant difference was observed, and white means this enzyme was not found in this study. The numbers in the upper half of the boxes correspond to EC numbers, and the numbers in the lower half correspond to the ratios of expression levels of these enzymes at 2, 5, 72, and 240 h compared with the control levels. 5.5.1.13: ent-copalyl diphosphate synthase; 4.2.3.19: ent-kaurene synthase; 1.14.13.78: ent-kaurene oxidase; 1.14.13.79: ent-kaurenoic acid oxidase; 1.14.11.12: gibberellin-44 dioxygenase; 1.14.11.15: gibberellin 3beta-dioxygenase; 1.14.11.13: gibberellin 2beta-dioxygenase; 1.14.13.90: zeaxanthin epoxidase; 5.3.99.9: neoxanthin synthase; 1.13.11.51: 9-cis-epoxycarotenoid dioxygenase; 1.1.1.288: xanthoxin dehydrogenase; 1.2.3.14: abscisic-aldehyde oxidase; 2.4.1.263: abscisate beta-glucosyltransferase; 3.2.1.21: beta-glucosidase; 2.5.1.27: adenylate dimethylallyltransferase; 2.5.1.75: tRNA dimethylallyltransferase

The expression levels of related enzymes involved in hormone synthetic pathways were also analyzed. The biosynthesis of ABA, ZR, and GA were all from mevalonate, which generates from terpenoid backbone biosynthesis (Fig. 3). The expression levels of most of the enzymes associated with the ABA biosynthetic pathway were up-regulated. For example, 9-cis-epoxycarotenoid dioxygenase (EC: 1.13.11.51; NCED), the rate-limiting enzyme that catalyzes the synthesis of xanthoxin by cleaving 9′-cis’-neoxanthinc and 9-cis-violaxanthin [40], increased 1.91-fold at 2 h (Fig. 3). Beta-glucosidase (EC: 3.2.1.21; GBA3), regulating the final conversion of ABA-glucose ester into bioactive ABA [41], increased 1.58-fold at 240 h. Zeaxanthin epoxidase (EC: 1.14.13.90; ZEP) and xanthoxin dehydrogenase (EC: 1.1.1.288; ABA2) also increased 2.80- and 1.95-fold at 240 h, respectively. Other enzymes, such as abscisic-aldehyde oxidase (EC: 1.2.3.14; AAO3), neoxanthin synthase (EC: 5.3.99.9; NSY), and abscisate beta-glucosyltransferase (EC: 2.4.1.263; AOG), displayed no significant change or were unidentified. Moreover, all enzymes related to the biosynthesis of GA and ZR had no differential expression or was unidentified. Gibberellin-44 dioxygenase (EC: 1.14.11.12; G44OX) and gibberellin 3-beta-dioxygenase (EC: 1.14.11.15; GA3OX) that participate in GA biosynthesis and cytokinin trans-hydroxylase (CYP735A) involved in ZR biosynthesis showed no differential change. Other key enzymes, such as ent-copalyl diphosphate synthase (EC: 5.5.1.13; CPS), ent-kaurene synthase (EC: 4.2.3.19; KS), and adenylate isopentenyltransferase (EC: 2.5.1.27; IPT) were unidentified.

The proteomic expression profile was consistent with the variation in hormones content of *L. punctata* after application of uniconazole. A previous study found that uniconazole regulates plant changes by inhibiting GA biosynthesis and ABA catabolism [42]. However, few studies have investigated the variation in these pathways in detail except for several reports on biochemical and physiological analyses [43]. There are C15 direct and C40 indirect pathways for ABA biosynthesis. Most ABA generates from C40 carotenoid through the indirect cleavage process in higher plants [44]. In our study, ABA in *L. punctata* is the most abundant hormone and its content increased to 2.7 times its original amount at 240 h, which is approximately 14-fold more than ZR and GA. Proteomic analysis of the ABA biosynthetic pathway also showed that most of the enzymes, including GBA3, ZEP, ABA2, and especially the key enzyme NCED, had up-regulated expression. On the other hand, the GA and ZR content always had a relatively low value. Similarly, these enzymes involved in the biosynthetic pathways of GA and ZR, such as CPS, KS, G44OX, and GA30X, showed no differential change or were not detected. The transcripts encoding these enzymes also had a relatively low level of expression.

### Starch accumulation of *L. punctata* under uniconazole treatment

**Fig. 4:**
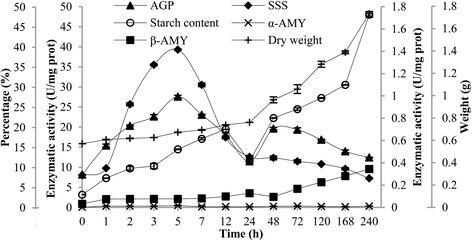
Dry weight, starch content, and activities of AGP, SSS, α- and β-AMY in duckweed. The starch content corresponds to the left of main Y-axis, activities of AGP and SSS correspond to the right of main Y-axis, activities of α- and β-AMY correspond to the left of minor Y-axis, and dry weight corresponds to the right of minor Y-axis

The dry weight and starch content increased significantly after application of uniconazole. According to the calculated average, the dry weight of *L. punctata* in one flask increased continuously from 0.57 to 1.74 g by 240 h (Fig. 4). Meanwhile, there was a significant increase in starch content from 3.16 to 48.01 % of DW by 240 h. The dry weight and starch content increased 3.1- and 15.2-fold, respectively. The activity of enzymes involved in starch biosynthesis and degradation was also analyzed. First, the activity of ADP-glucose pyrophosphorylase (EC: 2.7.7.27; AGP), the most important key enzyme in starch biosynthesis, increased from 8.20 to the maximum of 27.59 U/mg prot by 5 h (Fig. 4). Similarly, the activity of soluble starch synthetase (EC: 2.4.1.21; SSS), which promotes amylopectin biosynthesis, also increased from 8.03 to 39.29 U/mg prot by 5 h. Furthermore, starch can be degraded by two amylases into dextrin and maltose to supply energy for utilization in life activities. The activities of alpha-amylase (EC: 3.2.1.1; α-AMY) stabilized at a level of 0.003 U/mg prot. However, those of beta-amylase (EC: 3.2.1.2; β-AMY) increased from 0.032 to 0.345 U/mg prot by 240 h.

**Fig. 5:**
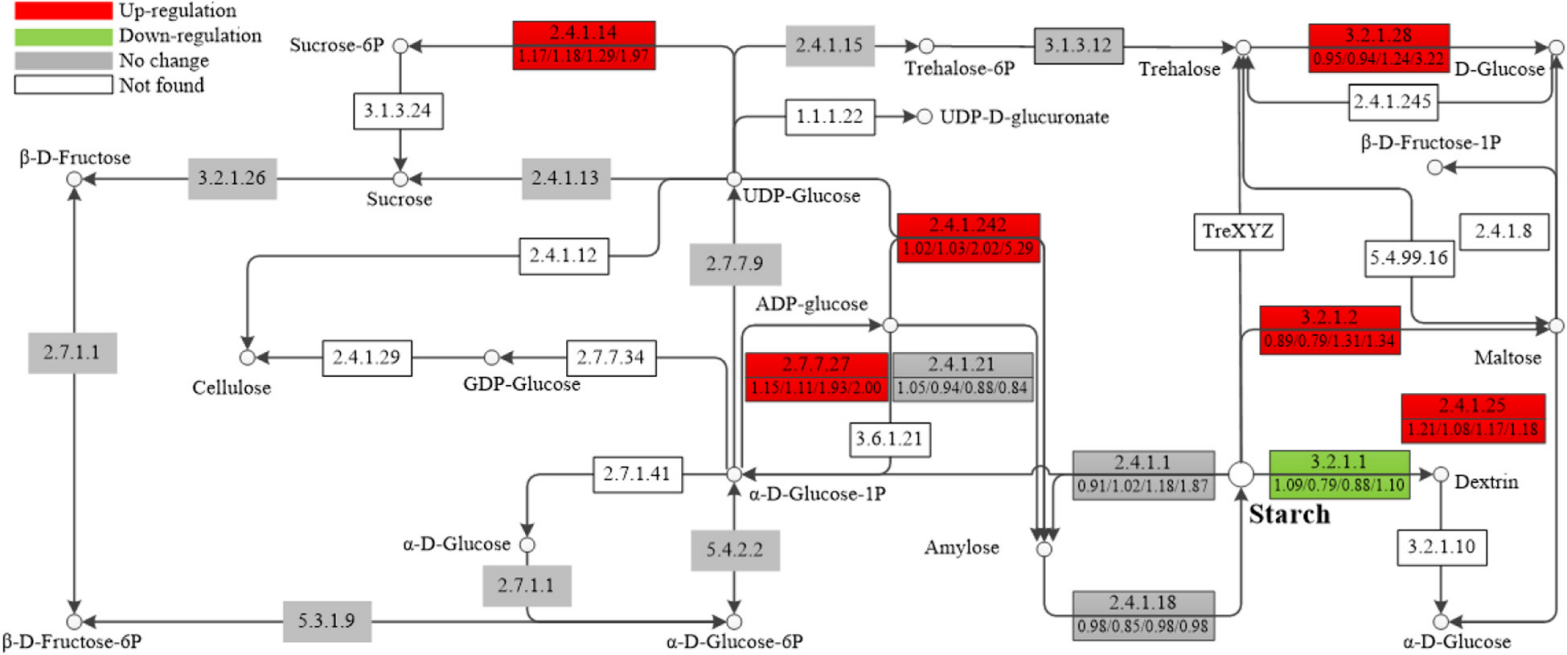
Expression patterns of some enzymes involved in carbon metabolism. Red boxes indicate the up-regulated enzymes in response to uniconazole, green for down-regulated, gray means no significant difference was observed, and white means this enzyme was not found in this study. The numbers in the upper half of the boxes correspond to EC numbers, and the numbers in the lower half correspond to the ratios of expression levels of these enzymes at 2, 5, 72, and 240 h compared with the control levels. 2.7.7.27: ADP-glucose pyrophosphorylase; 2.4.1.11: granule bound starch synthase; 2.4.1.21: soluble starch synthase; 2.4.1.1: glucan phosphorylase; 3.6.1.21: ADP-sugar diphosphatase; 2.4.1.18: starch branching enzyme; 3.2.1.1: alpha-amylase; 3.2.1.2: beta-amylase; 2.7.7.9: UDP-glucose pyrophosphorylase; 2.4.1.12: cellulose synthase; 2.4.1.13: sucrose synthase; 2.4.1.14: sucrose phosphate synthase; 3.1.3.24: sucrose-6-phosphate phosphatase; 2.4.1.25: 4-alpha-glucanotransferase; 2.4.1.15: trehalose-6-phosphate synthase; 3.1.3.12: trehalose 6-phosphate phosphatase; 3.2.1.28: trehalase; 3.2.1.26: beta-fructofuranosidase

The expression profiles of enzymes involved in the starch metabolic pathway were analyzed further. To begin with, two key enzymes related to starch biosynthesis, AGP and granule bound starch synthase (EC: 2.4.1.242; GBSS), displayed up-regulated expression (Fig. 5). AGP, the first key enzyme responsible for ADP-glucose synthesis and transfer, consists of two identical large subunits (AGP-LS) and two identical small subunits (AGP-SS). The AGP-LS and AGP-SS are in charge of regulatory function and catalytic activity, respectively [45]. GBSS, closely combined with starch granules, plays a key role in the biosynthesis of amylose. The results showed that AGP-LS and GBSS significantly increased 2.00- and 5.29-fold at 240 h, respectively. However, no significant increases were observed for other key enzymes or subunits, such as AGP-SS, soluble starch synthase (EC: 2.4.1.21; SSS) or starch branching enzyme (EC: 2.4.1.18; SBE). In addition, in the starch degradation process, α-AMY and β-AMY play the chief roles in plants. α-AMY displayed expression level decreases to 0.79 that of the control level at 5 h, but β-AMY increased 1.34-fold at 240 h. Finally, almost all enzymes involved in some other carbohydrate metabolic branches, which compete with starch biosynthesis for substrates to synthesize sucrose, cellulose and trehalose, shared no significant change or were unidentified. In sucrose biosynthesis, sucrose synthase (EC: 2.4.1.13; SuSy) displayed no significant change and sucrose-6-phosphate phosphatase (EC: 3.1.3.24; SPP) was not identified. However, sucrose-phosphate synthase (EC: 2.4.1.14; SPS) was identified and increased 1.97-fold at 240 h. Cellulose synthase (EC: 2.4.1.12; CESAs), which catalyzes cellulose biosynthesis, was not identified. Neither trehalose-6-phosphate synthase (EC: 2.4.1.15; TPS) nor trehalose-6-phosphate phosphatase (EC: 3.1.3.12; TPP), which are involved in trehalose biosynthesis, displayed significant differences.

The proteomic analysis, enzymatic assay and composition determination were combined to understand the rapid high starch accumulation observed after application of uniconazole. The data were analyzed and compared at three different levels. Starch composition determination showed that starch content increased rapidly. At 240 h, the starch percentage increased from 3.16 to 48.01 % (DW) (Fig. 4). The mean of total starch weight in one flask increased from 1.8 to 83.5 mg, which meant that the starch quantity increased to 46.4 times its origin amount. Meanwhile, the enzymatic activity assay also indicated that activities of AGP and SSS, key enzymes related to starch biosynthesis, increased 3.4 and 4.9 times, respectively. Both α-AMY and β-AMY had very low enzymatic activities at all samples, which were far below those of starch biosynthesis-related enzymes. Fig. 4 showed a dip in starch content and activities of AGP and SSS at 24 h. In this research, there was 8 h dark from 12 to 20 h, therefore, the dip may result from starch metabolism and lack of production in this period. Most importantly, proteomic data showed a very similar trend to the above mentioned results. On the one hand, uniconazole significantly up-regulated the expression levels of starch biosynthesis-related key enzymes, such as AGP-LS and GBSS (Fig. 5). On the other hand, the expression level of α-AMY was slightly down-regulated, but that of β-AMY was slightly up-regulated. In addition, there was no significant difference in the expression levels of regulatory enzymes involved in other carbohydrate metabolic branches that compete for glucose, except for SPS, which was increased. We deduced that many enzymes were undetected due to a very low abundance under the detection limit. The starch biosynthesis-related enzymes were highly expressed and up-regulated, but the degradation-related enzymes were hardly expressed and down-regulated. These results further demonstrated that high starch accumulation may have predominantly resulted from the synergistic effect of regulated expression of these enzymes in the relevant pathways under uniconazole application, which were consistent with the transcriptomic data, enzymatic assay, and composition determination.

So far, few studies have been conducted that systematically investigate the uniconazole-induced ABA, GA, and ZR biosynthesis and starch accumulation in plants. Uniconazole is well known as a plant growth retardant through inhibition of GA biosynthesis [25]. The effects of uniconazole on GA biosynthesis and metabolism in rice plants have been well established [46]. Many previous researches about uniconazole mainly focus on its effects on plant height [47], improved grain quality [30], and enhancement of tolerance to stress [48]. This integrated study provided much information about endogenous hormone changes and hormone-regulated starch accumulation induced by the chemical growth regulator.

**Fig. 6:**
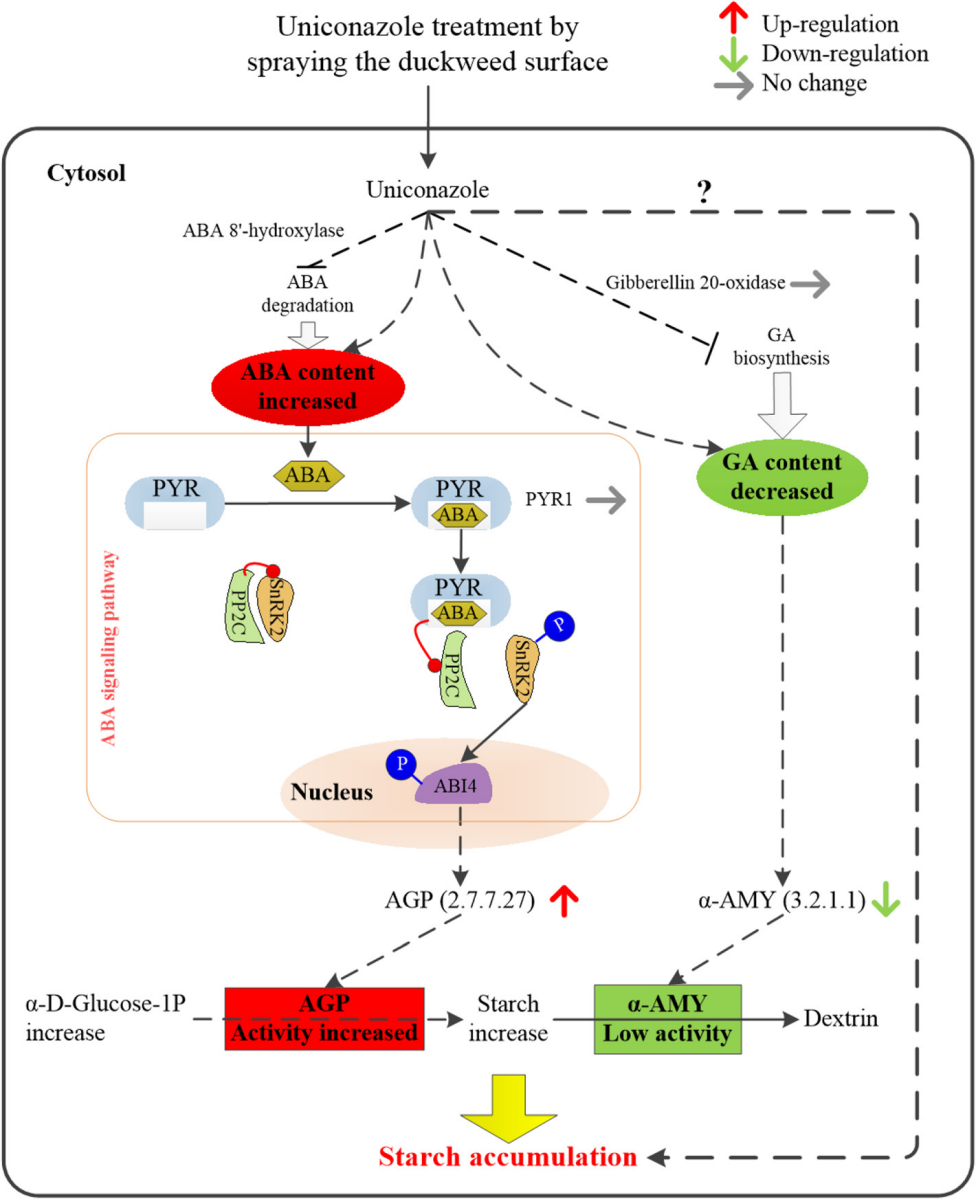
A proposed model in which ABA and other endogenous hormones modulate starch accumulation. Red upward arrow indicates up-regulated protein in response to uniconazole, green downward arrow for down-regulated, gray arrow means no significant difference was observed in this research. The model was constructed to help understanding uniconazole-induced hormone variation and starch accumulation in *L. punctata*. Uniconazole induces in expression of hormone-associated enzymes that result in changes in endogenous hormone contents. ABA is bound by PYRs and then initiates ABA signaling pathway [49, 50]. The significantly increased ABA and decreased GA modulate the expression of some enzymes involved in starch metabolism, and these enzymes finally result in starch accumulation. There may be an unknown regulation mechanism of starch accumulation, which is indicated by a question mark. See text for detailed description of the model. PYR: pyrabactin resistance; AGP: ADP-glucose pyrophosphorylase; α-AMY: alpha-amylase

To understand the uniconazole-induced starch accumulation in *L. punctata*, a putative model was constructed, in which ABA and other endogenous hormones modulate starch accumulation in response to uniconazole application (Fig. 6). The significantly increased endogenous ABA induced by uniconazole may play the leading role in regulating starch accumulation instead of GA. According to the literature [42], uniconazole, as a potential inhibitor of ABA catabolism, also contributes to the enhancement of ABA content. As shown in the model, recent research found that early ABA signal transduction depends on pyrabactin resistance (PYR) protein and the regulatory component of the ABA receptor-type 2C protein phosphatases-snf1-related protein kinase (PYR/RCAR-PP2C-SnRK2) pathway [49]. The PYRs are ABA receptors functioning at the apex of negative regulatory pathway that controls ABA signaling. First, ABA is bound by PYRs, which dissociate to form ABA receptor-PP2C complex to initiate ABA signaling pathway [50]. Therefore, complex formation inhibits the activity of the PP2C in an ABA-dependent manner, allowing activation of SnRK2s. Then, ABA-insensitive 4 (ABI4) is activated by phosphates of SnRK2, resulting in control of gene expression of *APLs* [51]. Finally, the transcription of *APLs* influences protein expression and enzymatic activity of ADP-glucose pyrophosphorylase (AGP). In this research, proteomic result showed that no significant difference was observed for the expression of PYR1 protein. AGP, a key enzyme in starch synthetic pathway, exhibited up-regulated expression and increased enzymatic activity, resulting in starch accumulation. Wang [52] also gained high-starch dormant bud structure (turions) of *S. polyrrihza* in ABA enhanced medium, but it took a long time and could hardly be produced in full scale. After ABA treatment, the genes of *APL2* and *APL3* of *S. polyrhiza* were highly expressed in earlier stages of turions development. Moreover, GA induced release of potato tuber bud dormancy and expression of α-amylase to mediate starch degradation [53]. Uniconazole-induced GA reduction may similarly induce low expression of α-amylase, which also contributed to accumulate high levels of starch. Additionally, there may be an unknown regulated mechanism of starch accumulation, which is indicated by a question mark (Fig. 6). The change in endogenous hormone levels induced by uniconazole, including ABA, GA and others, co-regulated the starch accumulation in *L. punctata*. This model interfaces with hormone change, signal transduction, protein expression, enzymatic activity, and starch accumulation, thus providing a mechanistic connection between the uniconazole application and starch accumulation.

## Conclusions

In summary, uniconazole has the potential to enhance the quantity and quality of duckweed feedstock for biofuel production. A proteomic approach was employed to investigate the response of duckweed after application of uniconazole. The results demonstrated that rapid accumulation of high levels of starch resulted from regulated expression of enzymes in the relevant pathways, which were consistent with the transcriptomic data, enzymatic assay, and composition determination. This study not only provided insights into the molecular mechanisms of uniconazole-induced hormone variation and starch accumulation but also highlighted the potential for duckweed to be feedstock for biofuel as well as for sewage treatment.

## Methods

### Duckweed growth conditions and uniconazole application

*Landoltia punctata* 0202, a wild duckweed, was collected from Sichuan province, China and stored in resource bank of duckweed in Chengdu Institute of Biology, Chinese Academy of Sciences. Ethics and standards of reporting in the research had been approved and consented from researchers and participants. Experimental research on duckweed plants including collection and utilization of the material had complied with institutional, national, and international guidelines. Duckweed was cultivated in a standard 1/6 Hoagland nutrient solution (pH 7.0) [54] in a commercial growth chamber (under a light intensity of 130 μmol photons m^−2^ s^−1^, a photoperiod of 25 °C for 16 h a day and 15 °C for 8 h a night, 80 % relative humidity). After 3 days, 6.0 g fresh weight of fronds were then transferred into a 1.0 L nutrient solution in a 1.0 L glass flask with uniconazole treatment over a period of 10 days under identical conditions. Uniconazole was dissolved in 10 % methanol and then diluted with water. A total of 5 ml aqueous solution containing 800 mg/L uniconazole (10 % methanol solution as a control) was applied as spraying the surface of a population after transferring. Uniconazole-P powder (S: R = 79:21) was manufactured by Sumitomo Chemical (Osaka, Japan). After applying uniconazole, plants were harvested at a total of 13 different time points (0, 1, 2, 3, 5, 7, 12, 24, 48, 72, 120, 168, and 240 h). For each point, three biological replicates were carried out independently. Moreover, these samples were aliquoted, immediately snap-frozen in liquid nitrogen and stored at −80 °C for proteomic analysis.

### Composition characterization and enzymatic activity assay

A small number of the collected samples were parch-dried at 70 °C for 48 h to determine the dry weight, as well as the starch content. The starch content was measured as glucose content in total sugar via HPLC (Thermo 2795, Thermo Corp, USA)-ELSD (All-Tech ELSD 2000, All-tech, Corp, USA), following the method of Zhang [4]. The starch content equals glucose content/1.1. The extraction, purification, and determination of endogenous levels of ABA, ZR, and GA using an indirect ELISA technique were performed as described by Wang [55] and Yang [56]. The activities of starch biosynthesis-related enzymes (AGP, SSS) and degradation-related enzymes (α-AMY, β-AMY) were measured using methods described by Nakamura and Hammond [57, 58].

### Protein extraction and digestion

Determination of time points for proteomic analysis is vital to elucidate the rapid starch accumulation in response to uniconazole application. Five time points (0, 2, 5, 72, and 240 h) were subjected to iTRAQ proteomic analysis according to the composition changes and enzymatic activity assay results.

For each sample, 0.5 g fronds was ground into a powder in liquid nitrogen and resuspended in lysis buffer (8 M urea, 4 % 3-[(3-cholamidopropyl) dimethylammonio]-1-propanesulfonate (CHAPS), 40 mM Tris–HCl) with 1 mM phenylmethanesulfonyl fluoride (PMSF) and 2 mM ethylenediaminetetraacetic acid (EDTA) at a ratio 1:5 (W/V). After 5 min of vigorous vortexing, dithiothreitol (DTT) was also added to make a final concentration of 10 mM. The cell mixture was subjected to sonication for 15 min and placed at ambient temperature for 30 min. The mixture was centrifuged for 60 min at 40,000 × *g* at 10 °C, and the supernatant was mixed well with 10 % pre-cooled TCA/acetone (1:4, v/v), followed by precipitation at −20 °C overnight. The samples were then centrifuged for 60 min at 40,000 × *g* at 10 °C. After the cell pellets were washed twice with cold acetone and dried, they were dissolved in digestion buffer (500 mM triethylammonium bicarbonate TEAB, 0.05 % w/v sodium dodecyl sulfate, SDS), aliquoted and stored at −80 °C. The protein concentration was determined using the Bradford method. After quantifying the concentration, equal aliquots (100 μg) from each sample were set up in a centrifuge tube and digested by incubation at 37 °C overnight with trypsin (Sigma; 1:20 w/w added at 0 and 4 h) and lyophilized.

### iTRAQ labeling, SCX fractionation and LC-ESI-MS/MS analysis

The iTRAQ labeling of digested peptide samples was performed following the manufacturer’s protocol with the iTRAQ® Reagent 8-plex Kit (AB SCIEX, USA). Five samples (0, 2, 5, 72, and 240 h) were labeled with iTRAQ tags 114, 115, 117, 119, and 121, respectively. The labeled peptides were then combined and vacuum centrifuged to dryness. After combining the four labeled samples, they were fractionated using strong cation exchange chromatography. The iTRAQ labeled peptides were dissolved in 4 mL of buffer solvent A (25 mM NaH_2_PO_4_, 25 % acetonitrile, pH 2.7), centrifuged for 20 min at 14,000 × *g* and loaded onto a Ultremex SCX column (250 × 4.6 mm, 5 μm) using an Agilent series 1100 HPLC instrument (Agilent technologies) at a flow rate of 1.0 mL/min. The column was first equilibrated in 95 % solvent A (mentioned above) for 10 min, and the following gradient elution program was then used: 5 % solvent B (25 mM NaH_2_PO_4_, 1 M KCl in 25 % acetonitrile, pH 2.7) for 7 min, 5–60 % B for 20 min, 60–100 % B for 2 min, and 100 % B for 1 min. A total of 20 fractions were collected based on the ultraviolet absorption with 214 nm. The collected fractions were desalted with StrataX column and concentrated to dryness using a vacuum centrifuge for LC-ESI-MS/MS analysis.

The LC-MS/MS analysis was performed using an AB SCIEX TripleTOF™ 5600 mass spectrometer (AB SCIEX, USA) coupled with a Tempo Nano HPLC system (AB SCIEX, USA). Each fraction was reconstituted in buffer A (5 % acetonitrile 0.1 %, formic acid) with a concentration of 0.5 μg/μl and then centrifuged for 10 min at 12,000 × *g* to obtain the supernatant. The fractioned peptides were separated with a nanobored C18 column with a picofrit nanospray tip (75 μm ID × 150 mm, 5 μm particles; New Objective, USA). Then, 5 μL fraction solutions were loaded onto the LC-Nano ESI-MS/MS system at a flow rate of 8 μl/min for 4 min with a splitter to obtain an effective flow rate of 0.3 μl/min. The peptides were eluted using the following procedure: 5 % solvent B (95 % acetonitrile, and 0.1 % formic acid) for 5 min, 5–35 % B for 35 min, 35–60 % B for 5 min, 60–80 % B for 2 min, 80 % B for 2 min, 80–5 % B for 1 min and 5 % B for 2 min. Peptides were electro-sprayed into the orifice of the mass spectrometer through coated silica tips at a high ion spray voltage of 2.5 kV. For MS data acquisition, the mass spectrometer was set to positive ion mode with a selected mass range 350–1250 m/z and TOF-MS accumulation time 0.25 s to detect precursor ions. Peptide ions with charge states 2^+^ to 4^+^ were selected for MS/MS. Information dependent acquisition of MS/MS was performed on the three most abundant peptides exceeding five counts were selected for MS/MS, and dynamic exclusion time was set at 30 s with ±30 mDa mass tolerance.

### Proteomic data analysis

The MS/MS data acquisition was performed with Analyst® QS 2.0 software (AB SCIEX). Raw data files acquired from the orbitrap were converted into MGF files. For protein identification, the data were processed by thoroughly searching against a database generated from our transcriptome data of *L. punctata* under uniconazole treatment using Mascot 2.3.02 (Matrix Science, London, United Kingdom) [59]. The transcriptome data were deposited in NCBI’s Transcriptome Shotgun Assembly (TSA) database under the accession number of PRJNA242298. Of the 140,432 contigs, 91,303 (65.0 %) got annotation information. For protein identification, the labeled peptides were automatically selected by the algorithm to calculate the reporter peak area, error factor and *p*-value. The relative abundances were determined using the reporter ion peak areas [60]. The parameters in the software were as follows: max missed cleavages, 1; enzyme, trypsin; cysteine alkylation, MMTS; species, plant; detected protein threshold, ±0.05 Da; peptide mass tolerance, 20 ppm; FDR determination of protein at 1 % FDR and distinct peptides at 5 %. Proteins with a fold change > 1.2 or <0.8 among different comparisons with a *p*-value less than 0.05 were considered to be differentially expressed proteins. Functional annotation of the proteins identified was performed using Blast2GO software, and GO categorization was achieved using WEGO [http://wego.genomics.org.cn/cgi-bin/wego/index.pl]. The KEGG pathway enrichment analysis of the responsive proteins was conducted according to the formula [61] from the KEGG Pathway Database [http://www.genome.jp/kegg/].

### Availability of supporting data

The transcriptome data set supporting the results of this article is available in the [NCBI’s Transcriptome Shotgun Assembly (TSA) database] repository, under the accession number of PRJNA242298 [unique persistent identifier and hyperlink to dataset in http://www.ncbi.nlm.nih.gov/gquery/?term=PRJNA242298]. The data set supporting the results of this article is included within the article.

## Additional files

Additional file 1:
**Up-regulated proteins after application of uniconazole.** The up-regulated proteins with >1.2-fold change and *p*-value less than 0.05 and their ratios among all comparative groups listed in the table. (XLSX 64 kb) Additional file 2:
**Down-regulated proteins after application of uniconazole.** The down-regulated proteins with <0.8-fold change and *p*-value less than 0.05 and their ratios among all comparative groups listed in the table. (XLSX 46 kb) Additional file 3:
**KEGG pathway analysis of the annotated proteins.** The pathway, number of annotated proteins, and pathway ID were listed in the table. (XLSX 15 kb)

## Abbreviations

DW: Dry weight
iTRAQ: Isobaric tags for relative and absolute quantification
LC-MS/MS: Liquid chromatography-tandem mass spectrometry
EC: Enzyme codes
GO: Gene Ontology
KEGG: Kyoto Encyclopedia of Genes and Genomes
ABA: Abscisic acid
CKs: Cytokinins
GAs: Gibberellins
